# Is Vitamin D Binding Protein a Novel Predictor of Labour?

**DOI:** 10.1371/journal.pone.0076490

**Published:** 2013-10-04

**Authors:** Stella Liong, Megan K. W. Di Quinzio, Gabrielle Fleming, Michael Permezel, Harry M. Georgiou

**Affiliations:** 1 Department of Obstetrics and Gynaecology, University of Melbourne, Parkville, Australia; 2 Mercy Perinatal Research Centre, Mercy Hospital for Women, Heidelberg, Australia; Baylor College of Medicine, United States of America

## Abstract

Vitamin D binding protein (VDBP) has previously been identified in the amniotic fluid and cervicovaginal fluid (CVF) of pregnant women. The biological functions of VDBP include acting as a carrier protein for vitamin D metabolites, the clearance of actin that is released during tissue injury and the augmentation of the pro-inflammatory response. This longitudinal observational study was conducted on 221 healthy pregnant women who spontaneously laboured and delivered either at term or preterm. Serial CVF samples were collected and VDBP was measured by ELISA. Binary logistic regression analysis was performed to assess the utility of VDBP as a predictor of labour. VDBP in the CVF did not change between 20 and 35 weeks' gestation. VDBP measured in-labour was significantly increased 4.2 to 7.4-fold compared to 4–7, 8–14 and 15–28 days before labour (*P*<0.05). VDBP concentration was 4.3-fold significantly higher at 0–3 days compared to 15–28 days pre-labour (*P*<0.05). The efficacy of VDBP to predict spontaneous labour onset within 3 days provided a positive and negative predictive value of 82.8% and 95.3% respectively (area under receiver operator characteristic curve  = 0.974). This longitudinal study of pregnant women suggests that VDBP in the CVF may be a useful predictor of labour.

## Introduction

Vitamin D binding protein (VDBP) is a 56–58 kDa plasma α-globulin that is primarily synthesised by hepatic parenchymal cells. Originally named “group-specific component” (Gc) [1], it was later identified as the major serum transporter protein for vitamin D and its metabolites, leading to the current nomenclature [2]. Circulating VDBP levels are stable in adult life [3]. There is no correlation between serum VDBP and the major circulating form and metabolite used routinely to assess overall vitamin D status, 25-hydroxyvitamin D (25-OHD) [4]. Characterisation studies have revealed VDBP to be highly polymorphic with over 120 variants identified [5]. The most common genetic variants of VDBP are the Gc1f, Gc1s and Gc2 isoforms and total circulating levels of VDBP may be dictated by the specific Gc phenotype [6].

VDBP has other physiological functions not restricted to vitamin D transportation. One of the more extensively studied properties is its high-affinity to form complexes with globular (G)-actin monomers, therefore inhibiting actin polymerisation [7], [8]. This rapid clearance of actin, released by damaged or lysed cells during tissue injury and inflammation, plays a major role in the prevention of downstream endothelial damaged caused by polymerised actin filaments. Indeed, VDBP displays a greater affinity towards actin monomers (1×10^9^ M^−1^) compared to vitamin D (25-OHD_3_: 7×10^8^ M^−1^ [9]; 1,25-(OH)_2_D_3_: 4×10^7^ M^−1^ [10]) underpinning its role in the inflammatory process. VDBP levels rise during the acute phase inflammatory response [11]. In addition to its actin-scavenging role, VDBP augments the chemotactic effect of C5a for neutrophil and macrophage migration in response to injury or inflammation [12], [13].

Aside from serum, VDBP has been detected in other biological fluids such as cerebrospinal fluid, urine and breast milk [14], [15]. A number of proteomic studies characterising human cervicovaginal fluid (CVF) have recently reported its presence among non-pregnant [16]–[18] and pregnant [19] women. Furthermore, studies utilising two-dimensional difference in-gel electrophoresis (2D-DIGE) techniques have demonstrated increased VDBP expression in association with spontaneous preterm birth [20] and preterm pre-labour rupture of the fetal membranes (preterm PROM) [21]. This raises the possibility that VDBP may be a potential biomarker of preterm birth, a condition that remains a global public health issue [22].

There are limited studies of VDBP in pregnancy. Bouillon and colleagues reported increased serum VDBP levels in pregnancy and in non-pregnant women using oestrogen therapy [4]. Other studies have also detected maternal VDBP on the cell-surface of human placental trophoblasts [23], [24]. The increase of VDBP, in particular VDBP:actin complexes in maternal serum during pregnancy, compared to non-pregnant women, may be from the high turnover of trophoblasts in the placental villous tissue that are in direct contact with maternal blood [25].

It is well established that both term and preterm labour are associated with increased inflammation and extensive tissue remodelling of the cervix and fetal membranes. As such, it is hypothesised that the VDBP concentration in the CVF may serve as an indicator of up-regulated cell death and tissue remodelling involved in cervical ripening and subsequent labour onset. Therefore the aims of this observational study were: (i) to investigate the concentration of VDBP in the CVF during the second half of pregnancy; (ii) to investigate the temporal VDBP concentration in the CVF of women with spontaneous term and preterm labour outcomes; and (iii) to determine the utility of VDBP to predict spontaneous labour onset.

## Materials and Methods

### Ethics

The collection of CVF from pregnant women was approved by the Mercy Health Human Research Ethics Committee (R06/56). All participants involved in this longitudinal observational study provided informed, written consent.

### Participants

Healthy, pregnant women attending the Mercy Hospital for Women, Heidelberg, Australia were recruited by a research midwife or medical officer. Exclusion criteria for recruitment were: obstetric complications such as hypertension, diabetes mellitus, known fetal anomaly, triplet or higher-order pregnancy; cervical cerclage *in situ*; or prescribed progesterone pessary treatment.

Following the aims described above, the proposed objectives required two arms of subject recruitment as it was deemed inappropriate to subject women to repeated frequent CVF sampling from 20 weeks' gestation until term labour onset. Women were either recruited preterm, in order to establish the antenatal expression of VDBP in the CVF and to optimise the number of samples from women who subsequently spontaneously laboured preterm, or recruited in late pregnancy in order to establish VDBP expression in the CVF in association with ‘normal’ term labour. Gestational age was calculated from first trimester ultrasound assessment. Specific details of recruitment are listed below.

### Preterm Recruitment

Women with either a singleton or twin pregnancy were recruited at 20–24 weeks' gestation, based on known risk-factor(s) for a preterm birth including: a history of spontaneous preterm labour; previous preterm pre-labour rupture of the fetal membranes; twin gestation; uterine anomaly; shortened cervix (<2.5 cm based ultrasonographic diagnosis); and a previous history of cervical cone biopsy. Recruited women had a CVF sample collected every four weeks until 35 completed weeks' gestation. Additional CVF samples were obtained from women presenting in spontaneous preterm labour, prior to rupture of the fetal membranes. The CVF samples were then retrospectively selected for analysis from women who experienced either spontaneous term labour or spontaneous preterm labour. All CVF samples were collected prior to any therapeutic modalities (e.g. maternal corticosteroids, tocolytics and antibiotics).

### Late-Pregnancy Recruitment

Women with a singleton pregnancy were recruited at approximately 36 weeks' gestation and had a CVF swab performed weekly and where possible, an in-labour swab was collected prior to rupture of the fetal membranes. CVF samples were then retrospectively selected for analysis from women who experienced spontaneous term labour. Labour was defined as regular painful contractions leading to subsequent effacement and dilatation of the cervix (3–7 cm).

At the time of sampling, all participants were asked whether they had had unprotected sexual intercourse in the preceding 48 h; experienced vaginal bleeding in the preceding 24 h; or had a transvaginal ultrasound or digital vaginal examination performed in the 6 h prior to CVF sample collection. Participants were also questioned on whether they were taking any medications (oral and/or vaginal).

Recruited women were subsequently excluded from analysis if they were diagnosed with bacterial vaginosis; presented with clinical signs of infection (e.g. chorioamnionitis, urinary tract infection); had a digital vaginal examination or transvaginal ultrasound within 6 h prior to CVF collection; had an induction of labour or an elective Caesarean section delivery.

### Sample collection and processing

All swabs were performed prior to the rupture of the fetal membranes in order to avoid amniotic fluid contamination of the CVF. CVF was collected from pregnant women during a routine speculum examination as previously described [26]. A dual-tipped swab was placed in the posterior fornix of the vagina for 30 s. The swab was then placed into 1 mL of chilled CVF extraction buffer (50 mM Hepes, 150 mM NaCl, 0.1% SDS, 1 mM EDTA, 1 mM Pefabloc SC 4-(2-aminoethyl)benzene sulfonyl fluoride (Roche Diagnostics GmbH, Mannheim, Germany)). Samples were processed immediately according to previously published methods and extracted CVF was stored at −80°C until required [26].

### Vaginal microbiology

Women recruited preterm had a high vaginal swab taken for microbiological assessment at the time of every CVF swab collection. Women recruited in late pregnancy had a single high vaginal swab taken on entry to the study. Microbiological assessment was performed according to standard laboratory methods.

### Vitamin D binding protein measurement

The VDBP concentration in the CVF was measured using the VDBP DuoSet® ELISA kit (R&D Systems, Minneapolis, MN) according to manufacturer's instructions. The linear detection range of the assay was 156.25 pg/mL to 1000 pg/mL. The sensitivity, inter-assay and intra-assay coefficients of variation for the assay were 23.86 pg/mL, 8.7% and 5.6%, respectively.

The VDBP concentrations presented in the Results section were normalised against the protein content of each CVF sample and are expressed as per milligram of protein. The total protein concentration was determined using the Bicinchoninic acid protein assay (Pierce Biotechnology, Rockford, IL).

### Statistical analyses

**Figure 1:**
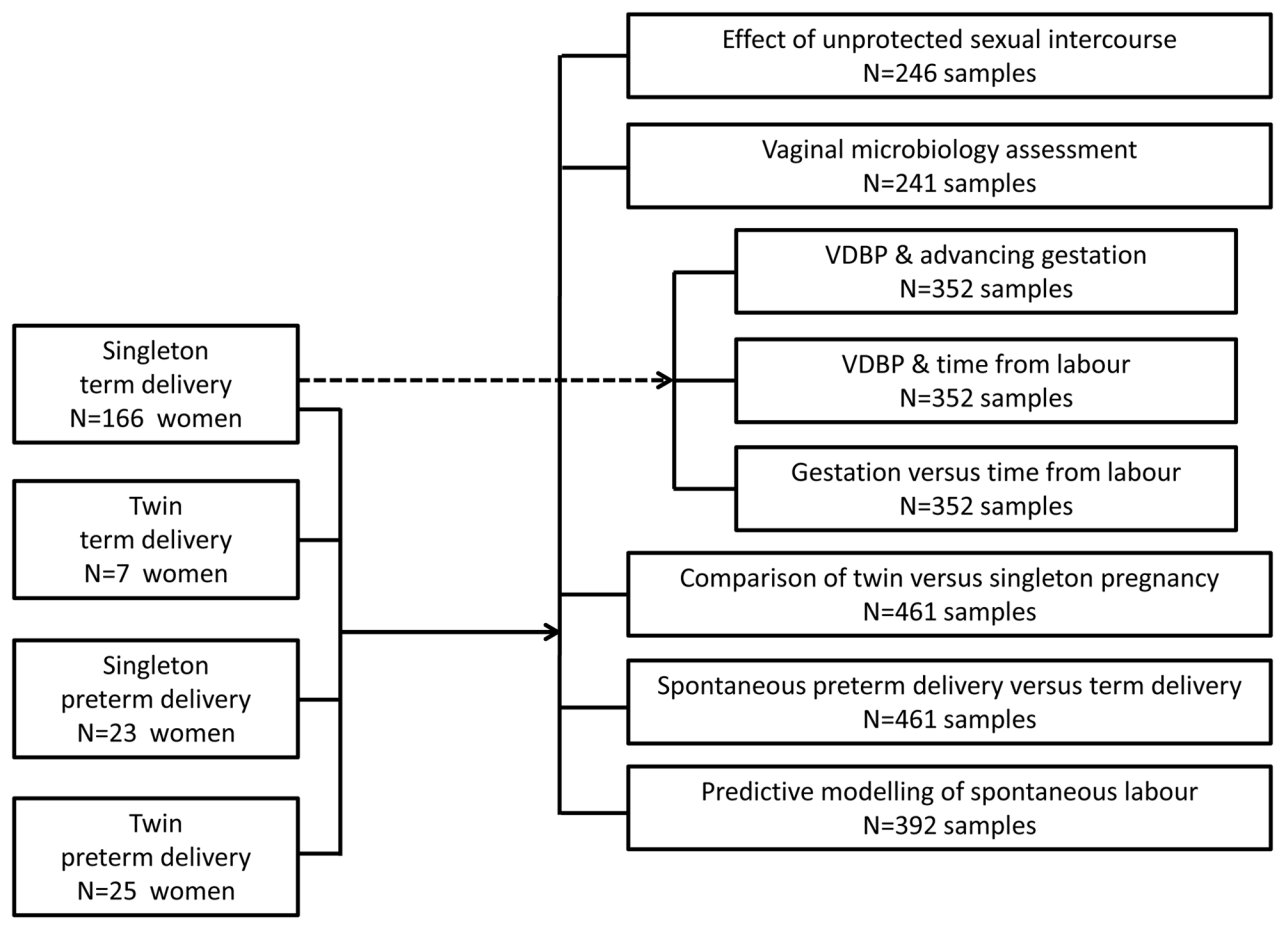
Flow Diagram illustrating the pool of 221 women recruited in the two arms of the study. Eight subanalyses of VDBP expression in the CVF were performed and the number of samples utilised is indicated.

From the 221 women recruited to the study, 461 CVF samples were collected. Subanalyses were performed according to the flow diagram represented in Figure 1. Statistical analyses were performed using the Statistical Package for Social Sciences (Version 20; SPSS Inc, Chicago, IL). Homogeneity of variance of protein-corrected VDBP data was not normally distributed (*P*<0.05, Kolmogorov-Smirnov test). Log transformation of the VDBP data was subsequently performed to normalise data. To account for multiple sampling, subjects were included as a random factor in statistical analyses, where appropriate. Comparisons between groups were performed using 2-way ANOVA with Tukey's Honestly Significant Difference (HSD) *post hoc* testing. Statistical significance was assumed when *P*<0.05.

Binary logistic regression was performed to assess the utility of VDBP as a predictive biomarker of spontaneous labour. Samples from women who spontaneously laboured either at term or preterm were used in the analysis. Only VDBP values from women who provided two or more CVF samples were utilised. To account for multiple sampling from the same subject, the subject was entered as a categorical variable. Receiver operator characteristic (ROC) curves were generated and the prediction of labour within 3, 7 and 14 days was determined. The sensitivity, specificity, positive predictive value (PPV) and negative predictive value (NPV) are reported based on a classification cut-off of 0.5.

## Results

### Demographics

A description of the participant demographics is summarised in Table 1. A total of 221 women participated in this study. Forty-eight women experienced subsequent spontaneous preterm labour (consisting of 23 women with a singleton pregnancy and 25 women with a twin pregnancy). The remaining 173 women subsequently spontaneously laboured at term (consisting of 166 singleton and 7 twin pregnancies).

**Table 1:** Demographic and clinical data of participants in the study (N = 221 women).

	Preterm labour	Term labour
	(N = 48 women)	(N = 173 women)
Maternal age (years)	32.28±5.1	31.69±4.6
Gravidity	N (%)	N (%)
1	11 (23%)	7 (4%)
2–3	28 (58%)	105 (61%)
≥4	9 (19%)	61 (35%)
Parity	N (%)	N (%)
Nulliparous	19 (40%)	27 (16%)
1	23 (48%)	103 (60%)
2–3	4 (8%)	36 (20%)
≥4	2 (4%)	7 (4%)
Singleton (N (%))	23 (48%)	166 (96%)
Twins (N (%))	25 (52%)	7 (4%)
Delivery gestation (weeks)	34.34±2.4	39.77±1.1
Baby weight (grams)	2289±595	3505±497

*Values are presented as mean ± SD*

### Sexual activity and vaginal microbiology

**Figure 2:**
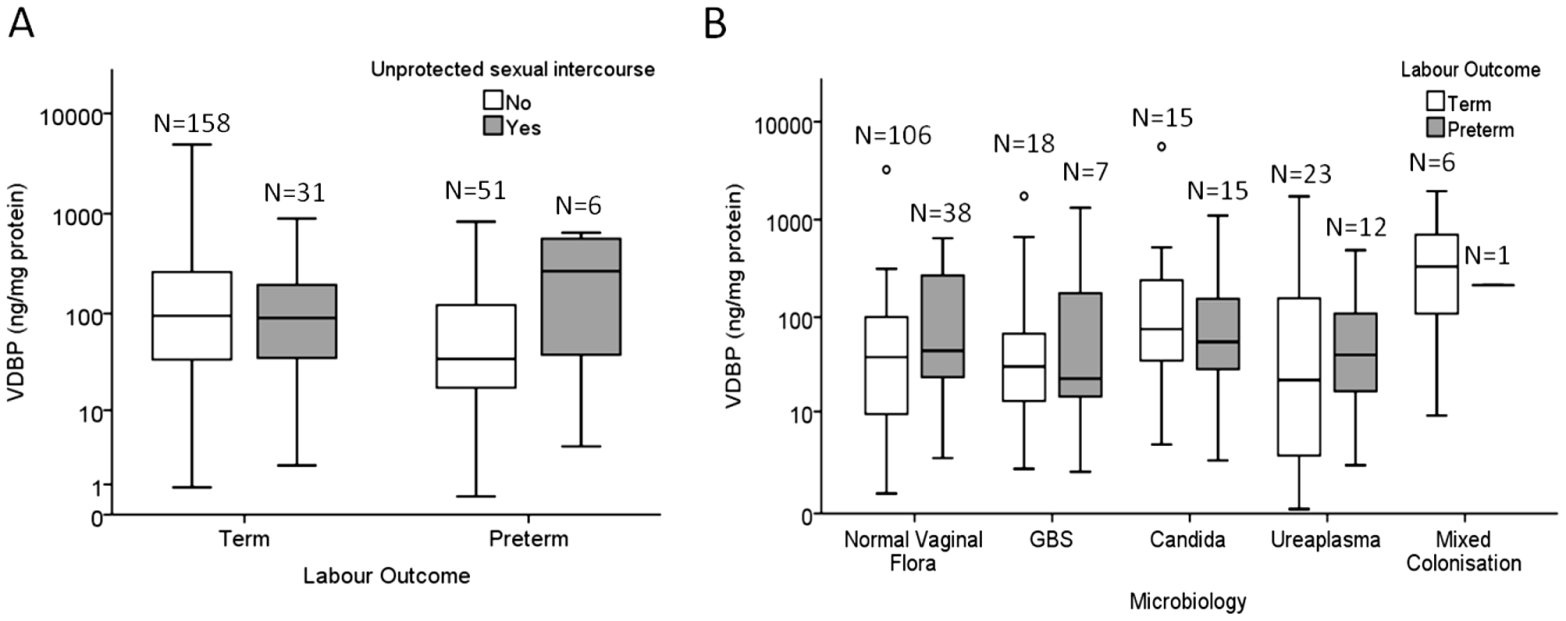
The effect of sexual intercourse and vaginal microbiology status on VDBP concentration. (A) Unprotected sexual intercourse within 48 h of CVF collection did not affect the VDBP concentration in women who delivered either term (*P* = 0.237) or preterm (*P* = 0.406, 2-way ANOVA). (B) The microbiology status of women with subsequent preterm or term labour did not affect the VDBP concentration. Within each labour group, VDBP was not significantly different between women with normal vaginal flora, Group B Streptococcus (GBS) colonisation, *Candida* spp. colonisation, *Ureaplasma* spp. colonisation, and women with mixed colonisation (Term: *P* = 0.478, Preterm: *P* = 0.557; 2-way ANOVA). The box and whisker plots represent the median and interquartile range with the 5^th^ and 95^th^ centile range. Outliers are represented by open circles.

Where the information was volunteered (53% of responses, N = 246 samples), the VDBP concentration was not significantly different in the samples obtained from women who had recent unprotected sexual intercourse compared with women who had not, irrespective of whether they delivered at term (N = 189 samples, *P* = 0.237) or preterm (N = 57 samples, *P* = 0.406, 2-way ANOVA, Figure 2A).

For analysis, the microbiology results were stratified into five groups: normal vaginal flora (NVF); NVF with Group B Streptococcus (GBS); NVF with *Ureaplasma* spp.; NVF with mixed colonisation (two or more of the above); and *Candida* spp. There was no significant difference in the VDBP concentration in the CVF of women with normal vaginal flora, Group B Streptococcus (GBS), *Ureaplasma* spp., mixed colonisation, or *Candida* spp., (N = 241 samples; Term outcome *P* = 0.478, Preterm outcome *P* = 0.557, 2-way ANOVA, Figure 2B).

### VDBP and gestation

**Figure 3:**
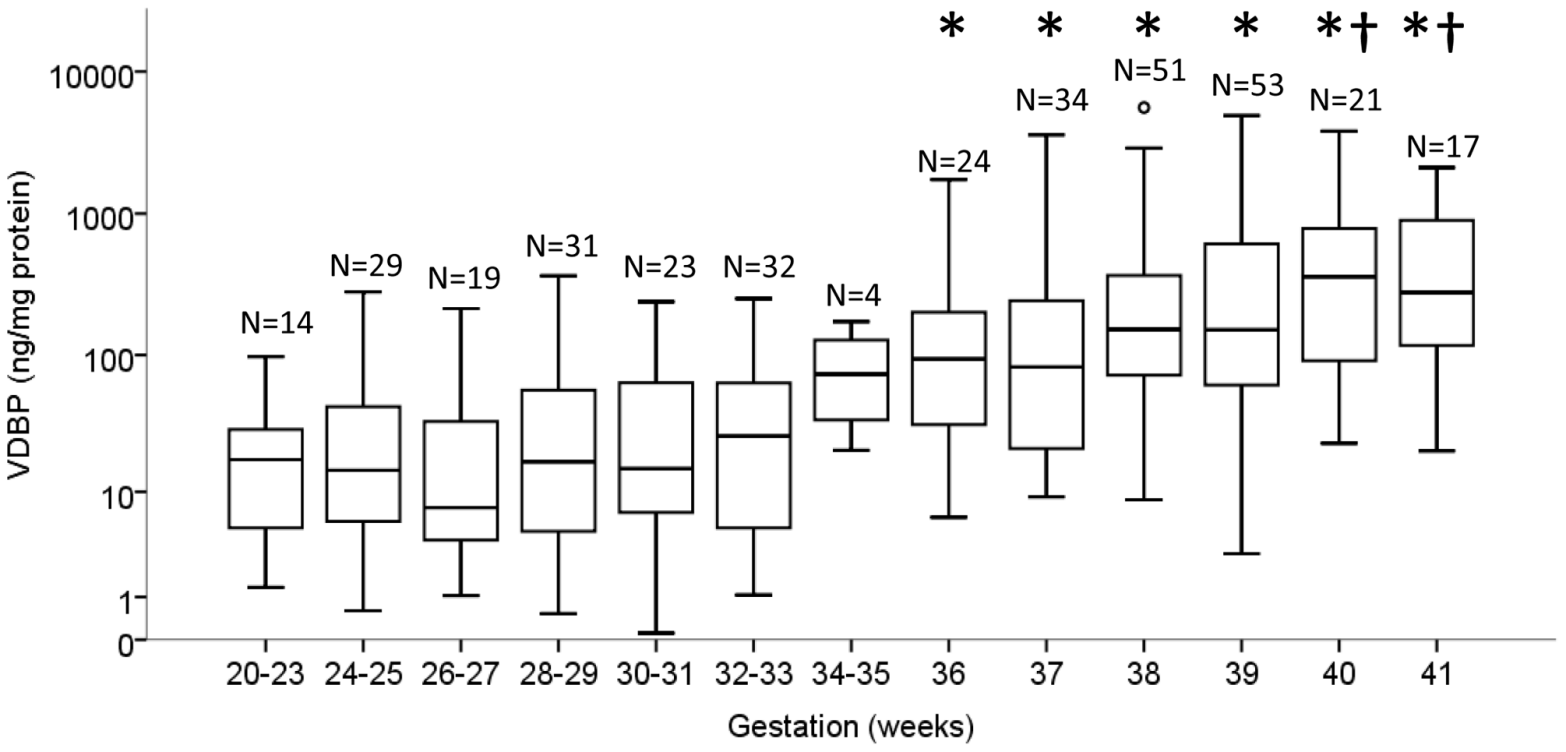
The VDBP concentration in the CVF significantly increased with advancing gestational age. The * indicates significance in the VDBP concentration in gestational groups ≤30–31 weeks' compared with CVF samples collected ≥36 weeks' gestation. The † indicates significance in VDBP concentration between samples collected at 36 and 37 weeks' gestation compared with samples collected at 40 and 41 weeks' gestation. Statistical significance was defined as *P*<0.05 (2-way ANOVA). The box and whisker plots represent the median and interquartile range with the 5^th^ and 95^th^ centile range. Outliers are represented by open circles.

A total of 352 samples were analysed to assess the effect of advancing gestation on the concentration of VDBP in the CVF. The VDBP concentration was not significantly different between gestational groups at 20–23, 24–25, 26–27, 28–29, 30–31, 32–33 and 34–35 weeks' gestation in women with a singleton pregnancy that subsequently reached term gestation (*P*>0.05, 2-way ANOVA, Figure 3). Samples collected after 36 weeks' gestation were significantly higher in VDBP compared to samples collected between 20–31 weeks' gestation (*P*>0.05, 2-way ANOVA). VDBP measured in women at 36 and 37 weeks' gestation was significantly lower compared to 40 weeks' (3.8 and 4.2-fold, respectively) and 41 weeks' gestation (3.0 and 3.4-fold, respectively) (all *P*<0.05, 2-way ANOVA). In contrast, there was no change in the VDBP concentration between 38–41 weeks' gestation (Figure 3).

### VDBP and term labour onset

**Figure 4:**
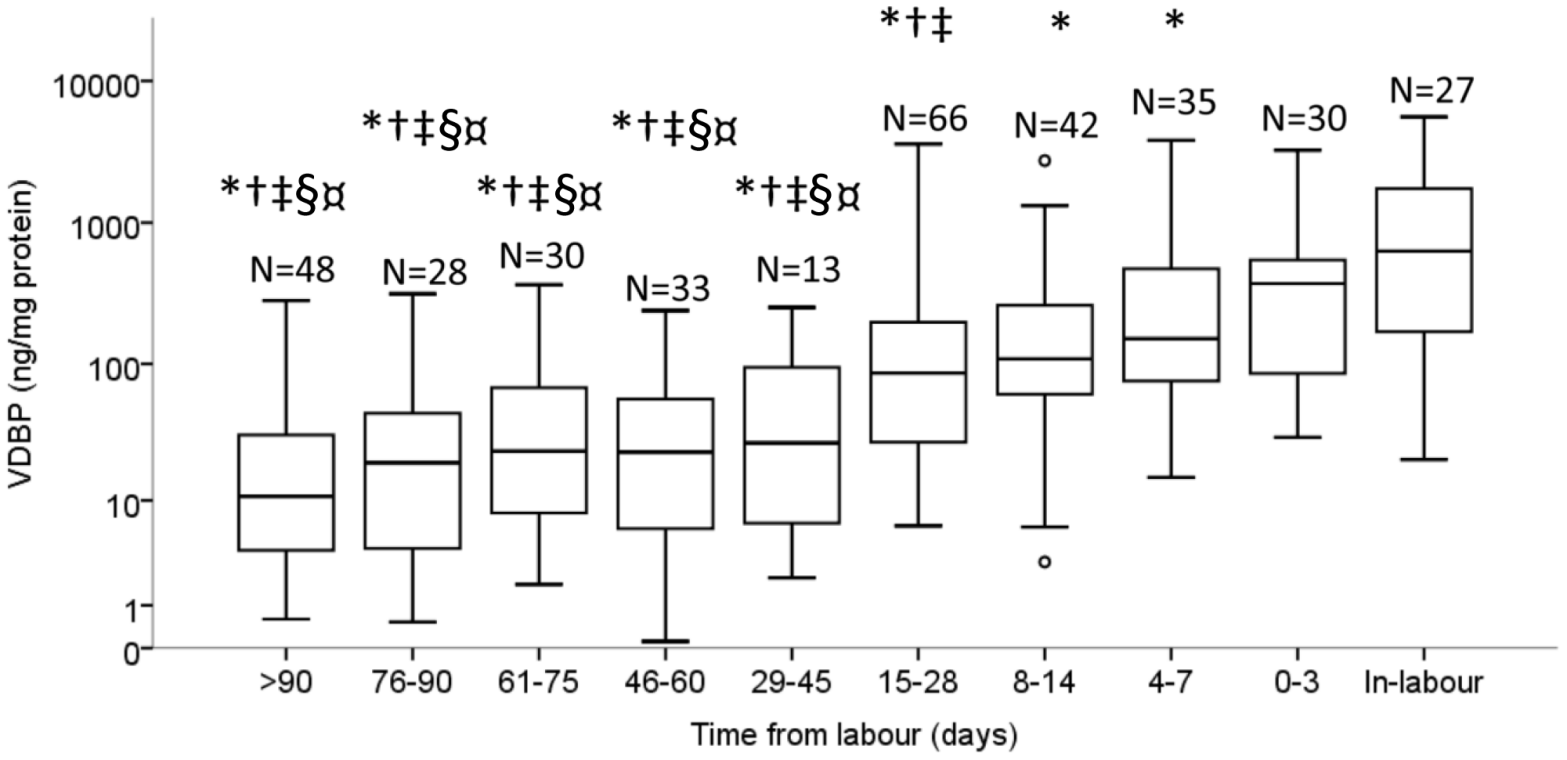
The VDBP concentration in the CVF significantly increased with approaching spontaneous term labour onset. The * indicates a significant difference in VDBP concentration in the CVF between the in-labour group and groups ≥4 days from labour. The † indicates a significant difference between the 0–3 day group versus groups ≥15 days from labour onset. The ‡ indicates a significant difference between the 4–7 day group versus groups ≥15 days from labour onset. The § indicates a significant difference between the 8–14 day group versus groups ≥29 days from labour onset. The ¤ indicates a significant difference between the 15–28 day group versus groups ≥29 days from labour onset. Statistical significance was defined as *P*<0.05 (2-way ANOVA). The box and whisker plots represent the median and interquartile range with the 5^th^ and 95^th^ centile range. Outliers are represented by open circles.

The same 352 samples described above were analysed with respect to the effect of time-from-labour on the concentration of VDBP. There was no significant change in the concentration of VDBP between 29 to >90 days prior to spontaneous term labour onset (*P*>0.05, 2-way ANOVA, Figure 4). By contrast, the VDBP concentration was significantly different with approaching labour in the final 28 days before spontaneous labour onset (*P*<0.05, 2-way ANOVA, Figure 4). The CVF concentration of VDBP was 4.2 to 7.4-fold significantly higher in-labour compared with samples collected at 4–7, 8–14 and 15–28 days pre-labour. VDBP at 0–3 days pre-labour was higher than at 8–14 days (3.4-fold) and significantly higher than at 15–28 days (4.3-fold) pre-labour. The VDBP concentration was also significantly elevated 1.8-fold at 4–7 days compared with samples collected 15–28 days pre-labour (Figure 4).

### Gestation versus time from labour

In women who delivered at term, the VDBP concentration increased both in terms of advancing gestation (Figure 3) and time from labour (Figure 4). In order to dissect the relative contribution of these two concurrent events, multivariate analysis on the 352 samples indicated that VDBP is influenced by approaching labour (*P* = 0.042) and not by advancing gestational age (*P* = 0.987, 2-way ANOVA). In the final 28 days, the correlation of VDBP concentration with approaching labour was greater (*P*<0.001, Pearson's r = 0.376), compared with advancing gestation (*P*<0.001, r = 0.239).

### VDBP and preterm birth

**Figure 5:**
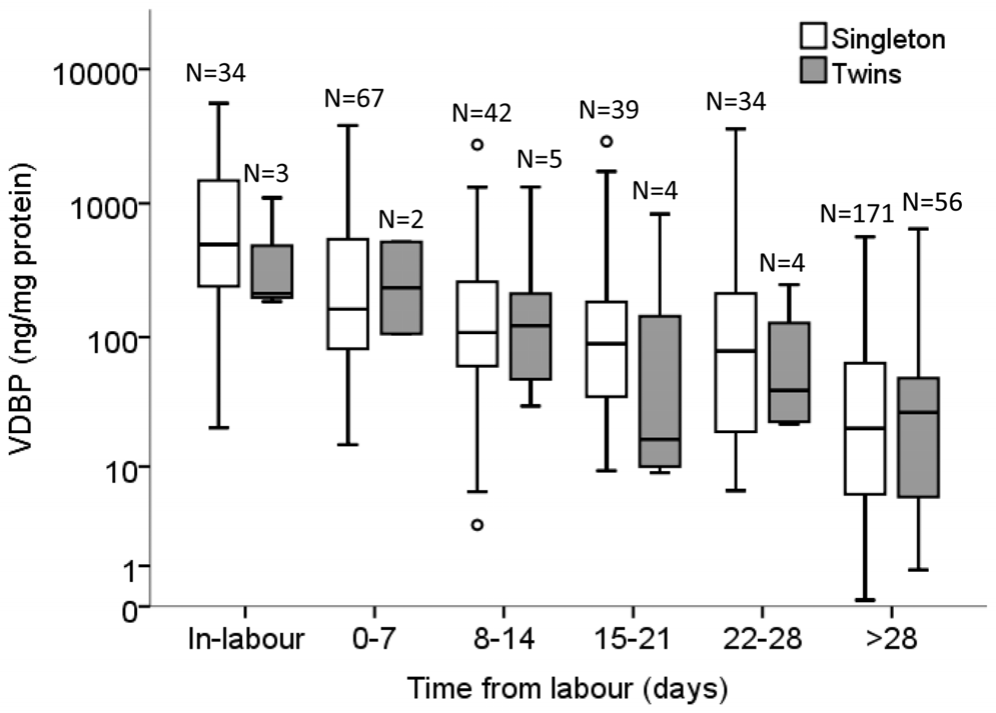
The comparison of VDBP concentration between singleton and twin pregnancies. VDBP was not significantly different between singleton and twin gestation in-labour (*P* = 1.000), 0–7 days (*P* = 0.997), 8–14 days (*P* = 1.000), 15–21 days (*P* = 0.750), 22–28 days (*P* = 0.999), and >28 days (*P* = 1.000) before labour onset (2-way ANOVA). The box and whisker plot represents the median and interquartile range with the 5^th^ and 95^th^ centile range. Outliers are represented by open circles.

To investigate VDBP expression in the CVF of women who delivered *preterm*, both singleton and twin pregnancies were included. Although the twin sample sizes were small in the <28 day time frame, the potential impact of twin gestation on the VDBP concentration in the CVF was determined. Figure 5 demonstrates that there was no significant difference in the VDBP concentration between women with a twin or singleton gestation at equivalent time frames from spontaneous labour (both term and preterm deliveries, N = 461 samples, *P*>0.05, 2-way ANOVA).

**Figure 6:**
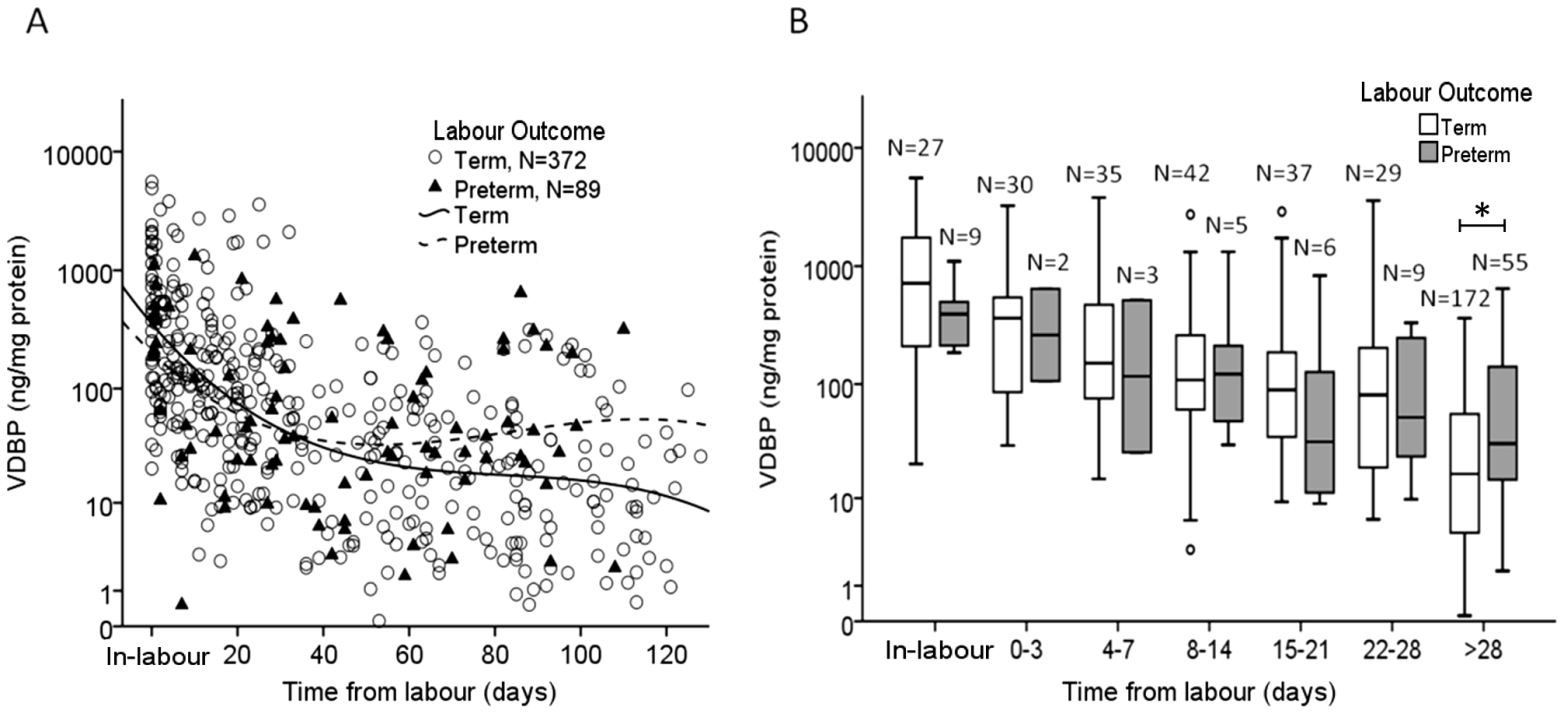
The comparison of VDBP concentration between term and preterm labour outcomes. (A) Women with *preterm* labour outcomes (dashed line) had an overall higher ‘baseline’ concentration of VDBP in the CVF collected >40 days before labour compared to women who ultimately delivered at term (solid line). (B) At each specified time point up to 28 days, there was no significant difference in the VDBP concentration between women who delivered at term and preterm. The * indicates a significantly increased concentration of VDBP in samples collected from women >28 days from labour who subsequently delivered *preterm* compared to samples from women >28 days from labour who delivered at *term* (*P*<0.001, 2-way ANOVA). The box and whisker plot represents the median and interquartile range with the 5^th^ and 95^th^ centile range. Outliers are represented by open circles.

Using the same 461 samples, a comparison of VDBP concentration between women who laboured at term and preterm was performed. Women with *preterm* labour outcomes were found to have an overall higher ‘baseline’ concentration of VDBP in the CVF collected between 40–125 days before labour compared to women who ultimately delivered at term (Figure 6A). A comparable level of expression was apparent in the final 4 weeks before spontaneous labour (Figure 6B) whether it be at *term* or *preterm* (*P*>0.05, 2-way ANOVA). CVF samples collected at >28 days from labour showed significantly increased VDBP concentration in women who experienced preterm labour compared to women with term birthing outcomes (*P*<0.001, 2-way ANOVA, Figure 6B).

### Utility of VDBP to predict labour

**Figure 7:**
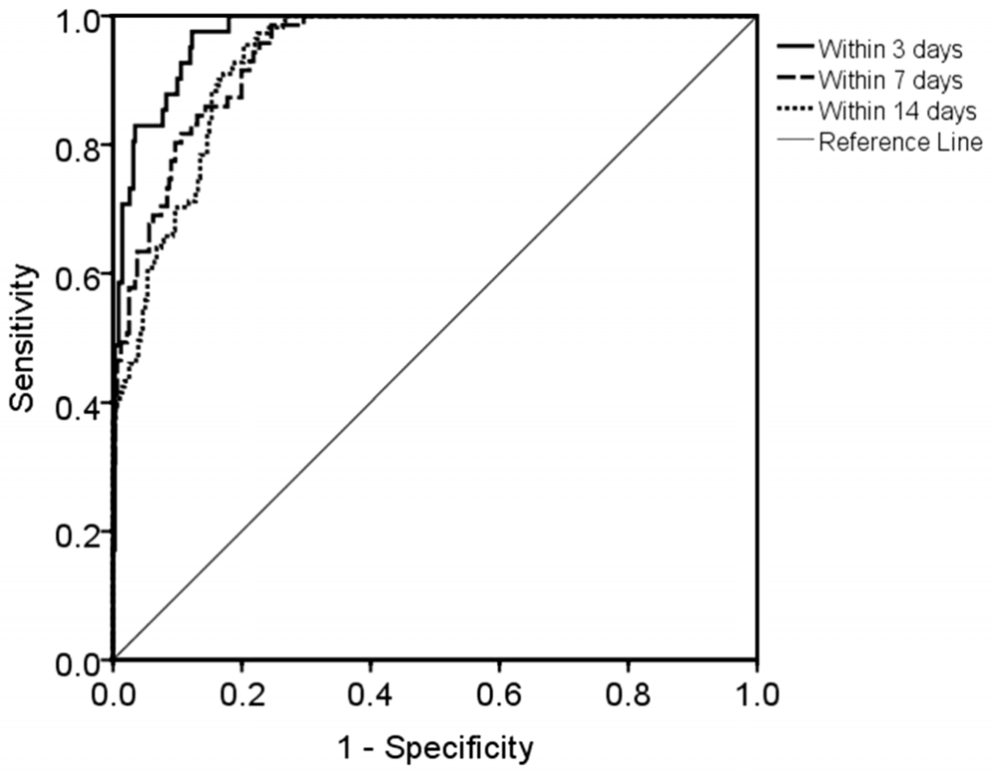
ROC curves of VDBP to predict labour onset within 3, 7 and 14 days. Subjects who provided two or more samples were included in the analysis (N = 392), with the subject entered as a categorical factor in the model. Area under the curves were 0.974 at ≤3 days; 0.943 at ≤7 days; and 0.934 at ≤14 days prior to spontaneous labour onset. Optimal predictive utility was obtained at ≤3 days from spontaneous labour.

To assess the utility of VDBP to predict labour up to 14 days before onset, a total of 141 women representing 392 samples were analysed. To take into account the relatively wide variation in VDBP concentration between women, only women who provided two or more CVF samples were analysed by Binary Logistic Regression. Samples from both term and preterm deliveries were included, as these demonstrated no significant difference in this time frame (Figure 6B). ROC curves were generated using the predicted probabilities obtained by binary logistic regression analyses to predict spontaneous labour up to 3, 7 and 14 days after sampling. The efficacy of VDBP to predict labour onset within 3 days after sampling provided an area under the curve of 0.974, *P*<0.001 (Figure 7) with 58.5% sensitivity, 98.6% specificity, 82.8% PPV and 95.3% NPV (Table 2). By comparison, the prediction of labour within 7 days yielded a sensitivity of 57.7% with PPV of 78.8%. Prediction within 14 days of labour provided a sensitivity of 71.2% and PPV of 69.9%.

**Table 2:** Efficacy of VDBP to predict spontaneous labour onset (N = 141 women; 392 samples).

Prediction	3 d before onset	7 d before onset	14 d before onset
Samples (N)	≤3 days; N = 41	≤7 days; N = 71	≤14 days; N = 111
	>3 days; N = 351	>7 days; N = 321	>14 days; N = 281
Area under curve	0.974	0.943	0.934
95% confidence interval	0.958–0.989	0.921–0.966	0.912–0.956
*P* value	<0.001	<0.001	<0.001
Classification cut-off value	0.5	0.5	0.5
Sensitivity (%)	58.5	57.7	71.2
Specificity (%)	98.6	96.6	87.9
Positive Predictive Value (%)	82.8	78.8	69.9
Negative Predictive Value (%)	95.3	91.2	88.5

## Discussion

To our knowledge, this is a novel study characterising the temporal changes of VDBP in the CVF in association with pregnancy and spontaneous labour. The presence of VDBP in the CVF during pregnancy is thought to be the result of plasma transudate. In the final four weeks of a term singleton pregnancy, we found that the VDBP concentration in the CVF was significantly elevated at 40–41 weeks' compared to 36–37 weeks' gestation (Figure 3). In addition we have found that beyond 37 weeks' gestation, the CVF concentration of VDBP significantly increases independent of gestation, suggesting that VDBP increases in association with approaching labour. Our preterm data also supports this finding.

The significant increase of VDBP in the CVF collected in-labour and at 0–3 days pre-labour may be attributed to the increased permeability of blood vessels as a result of the inflammatory process of labour leading to cervical remodelling and subsequent fetal membrane rupture. Indeed, synthesis of VDBP can be augmented by the presence of the pro-inflammatory cytokine IL-6, *in vitro* [27]. VDBP can associate with the surface of inflammatory cells [28], [29] as well as fibroblasts and smooth muscle cells [23]. Fibroblasts play a crucial role in the extracellular remodelling of the cervix, which is activated by the inflammatory process as the cervix ripens in preparation for labour [30].

The CVF expression of VDBP was similar in women who spontaneously laboured at term and preterm. In particular, the most dynamic change occurs in the final 4 weeks of pregnancy in both term and preterm cases. A significantly increased concentration of VDBP was found in samples collected from women >28 days from labour who spontaneously delivered preterm, compared to women who delivered at term (Figure 6A and 6B). This is an interesting observation and may indicate that women destined for a preterm birth have a higher ‘baseline’ expression of VDBP in the CVF.

There have been no previous temporal studies of VDBP expression in the CVF during pregnancy. A study conducted in Belgium by Bouillon *et al*, examined serum VDBP levels, serum vitamin D and seasonal factors in healthy, non-pregnant and pregnant women who were not taking vitamin D supplementation [31]. No seasonal variation was detected in VDBP in maternal serum. VDBP in the serum was increased during pregnancy compared to non-pregnant women and the concentration peaked between 32 to 35 weeks' gestation. However in Bouillon's study, the serum samples measured at 35±1 weeks' gestation were obtained from women at the time of preterm delivery. The authors did not take into account the ‘time from labour’ as a potential confounder. For women who subsequently laboured and delivered at term, we found that the VDBP concentration in the CVF did not change between 20 and 35 weeks' gestation.

The aetiology of spontaneous preterm birth is multifactorial. To strengthen this study, we have attempted to account for various potential confounders including risk factor status, vaginal flora, recent sexual activity, twin gestation and time from spontaneous labour. For women who delivered at term (both singleton and twin pregnancies), unprotected intercourse had no effect on the VDBP concentration in the CVF. For women who delivered preterm, again no difference was observed, however the sample size was underpowered to confirm this finding with confidence.

Multifetal gestation is a risk factor for preterm birth and this study included a proportion of women with a twin gestation. We recognise this is a limitation of the study however we included twin pregnancies in order to increase the preterm sample size. The increased VDBP concentration with respect to spontaneous labour onset (i.e. time from labour) was achieved using data derived from singleton pregnancies. We subsequently compared singleton and twin groups and found no significant difference in the VDBP concentration (both term and preterm outcomes) although twin sample sizes were small, particularly those less than 28 days from labour (Figure 5). A larger sample size of women with a twin pregnancy is needed to confirm these observations.

On the basis of the promising preterm data the utility of VDBP to predict spontaneous labour was investigated. The prediction of spontaneous labour within both 3 and 7 days compares favourably with fetal fibronectin (fFN). fFN remains the gold standard test for the prediction of labour [32], [33]. In clinical practice, the utility of fFN lies in its negative predictive value. The VDBP concentration in the CVF is not affected by recent unprotected sexual intercourse, which is a limitation of the fFN test. These findings highlight the robustness of VDBP as a potential biomarker of labour onset. We acknowledge that the predictive model in this study is limited and relies upon the serial sampling (minimum two) of CVF from the same woman. Unlike fFN, which is absent in the CVF at a preterm gestation, VDBP is ubiquitously expressed throughout pregnancy with a relatively wide variation between individuals. Further studies investigating the correlation of VDBP concentration with other clinical biomarkers of preterm labour (i.e. fFN and sonographic cervical length) is warranted and may improve the utility to predict spontaneous labour onset.

The role of VDBP and the contribution of its various isoforms in pregnancy is not fully understood. Activated B and T lymphocytes secrete enzymes that are able to process the O-linked side chain of VDBP [34]. This deglycosylated form of VDBP, also known as Gc-MAF, plays an important role in macrophage activation and differentiation. The efficiency of macrophage activation during an inflammatory response is also dependent upon different isoforms of VDBP [35]. The Gc2 isoform has only one glycosylation site compared to the Gc1f and Gc1s isoforms which carry two sites [36]. It is proposed that the Gc2 isoform has a reduced capacity to activate macrophages due to its partial deglycosylation and may support a link between VDBP polymorphism and increased susceptibility to certain diseases such as osteopetrosis and chronic obstructive pulmonary artery disease [37], [38]. In this context, a subset of women who experience spontaneous preterm birth may have a specific VDBP polymorphism. Indeed, further investigation of VDBP in maternal serum with respect to labour is warranted.

In conclusion, we have characterised VDBP in the CVF from mid- to late-pregnancy and report a significant increase in the final four weeks of pregnancy. Increased concentration of VDBP in the CVF was associated with spontaneous labour, both at term and preterm. The increase in VDBP may be indicative of increased inflammation and tissue damage that are integral to cervical remodelling and fetal membrane weakening with approaching labour onset. VDBP was able to predict spontaneous labour onset with high positive and negative predictive values up to 14 days from sampling. Further investigation to include other biomarkers (e.g. cervical length, fFN, IL-1ra [39]) may further enhance our ability to predict labour.
